# A monoclinic polymorph of 1-benzoyl-4-thio­biuret

**DOI:** 10.1107/S1600536813030250

**Published:** 2013-11-13

**Authors:** Namhun Kim, Haneol Kim, Sung Kwon Kang

**Affiliations:** a Department of Chemistry, Chungnam National University, Daejeon 305-764, Republic of Korea

## Abstract

The title compound, C_9_H_9_N_3_O_2_S, is a monoclinic (*C*2/*c*) polymorph of the previously reported triclinic structure [Kang (2013 ▶). *Acta Cryst.* E**69**, o1327]. The mol­ecule is almost planar with an r.m.s. deviation of 0.069 Å from the mean plane of all non-H atoms. The benzoyl and terminal thio­urea fragments adopt a *transoid* conformation with respect to the central carbonyl O atom. Two intra­molecular N—H⋯O hydrogen bonds are present. In the crystal, N—H⋯O and N—H⋯S inter­actions link the mol­ecules into zigzag chains extending along the *c-*axis direction.

## Related literature

For the biological activity of thia­diazole derivatives, see: Piskala *et al.* (2004 ▶); Castro *et al.* (2008 ▶). For the structure and reactivity of thia­diazole derivatives, see: Cho *et al.* (1996 ▶). For the structure of a thio­biuret compound, see: Kang *et al.* (2012 ▶) and of the monoclinic polymorph, see: Kang (2013 ▶).
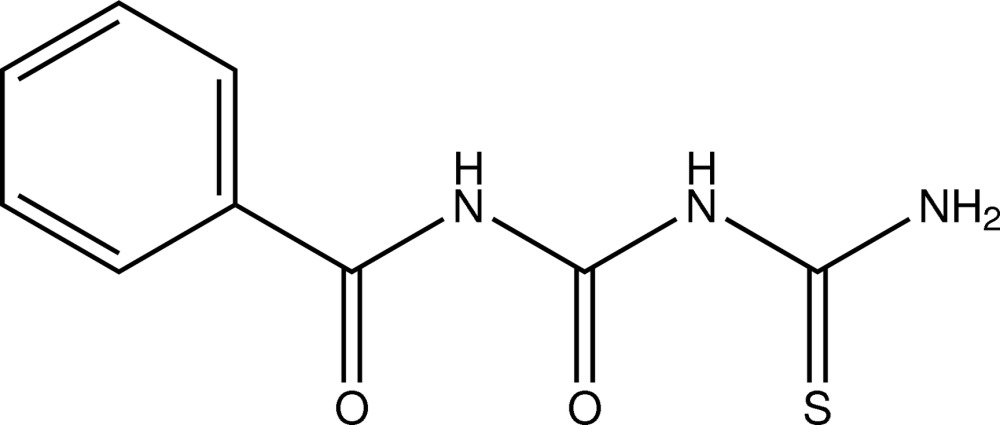


## Experimental

### 

#### Crystal data

C_9_H_9_N_3_O_2_S
*M* *_r_* = 223.25
Monoclinic, 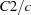

*a* = 14.4259 (14) Å
*b* = 6.6145 (6) Å
*c* = 21.722 (2) Å
β = 94.166 (3)°
*V* = 2067.2 (3) Å^3^
*Z* = 8
Mo *K*α radiation
μ = 0.30 mm^−1^
*T* = 296 K
0.18 × 0.12 × 0.04 mm

#### Data collection

Bruker SMART CCD area-detector diffractometer
Absorption correction: multi-scan (*SADABS*; Bruker, 2002 ▶) *T*_min_ = 0.96, *T*_max_ = 0.99
7756 measured reflections
1973 independent reflections
1293 reflections with *I* > 2σ(*I*)
*R*_int_ = 0.059

#### Refinement

*R*[*F* ^2^ > 2σ(*F* ^2^)] = 0.047
*wR*(*F* ^2^) = 0.115
*S* = 1.03
1973 reflections
152 parameters
H atoms treated by a mixture of independent and constrained refinement
Δρ_max_ = 0.21 e Å^−3^
Δρ_min_ = −0.23 e Å^−3^

### 

Data collection: *SMART* (Bruker, 2002 ▶); cell refinement: *SAINT* (Bruker, 2002 ▶); data reduction: *SAINT*; program(s) used to solve structure: *SHELXS2013* (Sheldrick, 2008 ▶); program(s) used to refine structure: *SHELXL2013* (Sheldrick, 2008 ▶); molecular graphics: *ORTEP-3 for Windows* (Farrugia, 2012 ▶); software used to prepare material for publication: *WinGX* (Farrugia, 2012 ▶).

## Supplementary Material

Crystal structure: contains datablock(s) global, I. DOI: 10.1107/S1600536813030250/lr2116sup1.cif

Structure factors: contains datablock(s) I. DOI: 10.1107/S1600536813030250/lr2116Isup2.hkl

Supplementary material file. DOI: 10.1107/S1600536813030250/lr2116Isup3.cml

Additional supplementary materials:  crystallographic information; 3D view; checkCIF report

## Figures and Tables

**Table 1:** Hydrogen-bond geometry (Å, °)

*D*—H⋯*A*	*D*—H	H⋯*A*	*D*⋯*A*	*D*—H⋯*A*
N9—H9⋯O11^i^	0.81 (3)	2.20 (3)	2.945 (3)	151 (2)
N12—H12⋯O8	0.86 (3)	1.97 (3)	2.661 (3)	136 (2)
N15—H15*A*⋯S14^ii^	0.98 (4)	2.41 (4)	3.358 (3)	163 (3)
N15—H15*B*⋯O11	0.81 (3)	2.06 (3)	2.653 (3)	130 (3)

Symmetry codes: (i) 
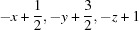
; (ii) 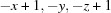.

**Fig. 1.**
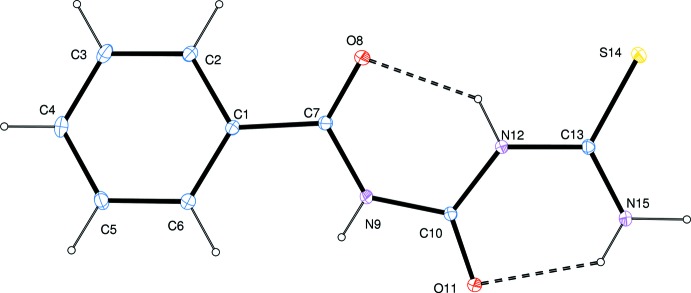
Molecular structure of (I) with ellipsoids drawn at the 30% probability level . Two intramolecular N—H···O hydrogen bonds are indicated by dashed lines.

**Fig. 2.**
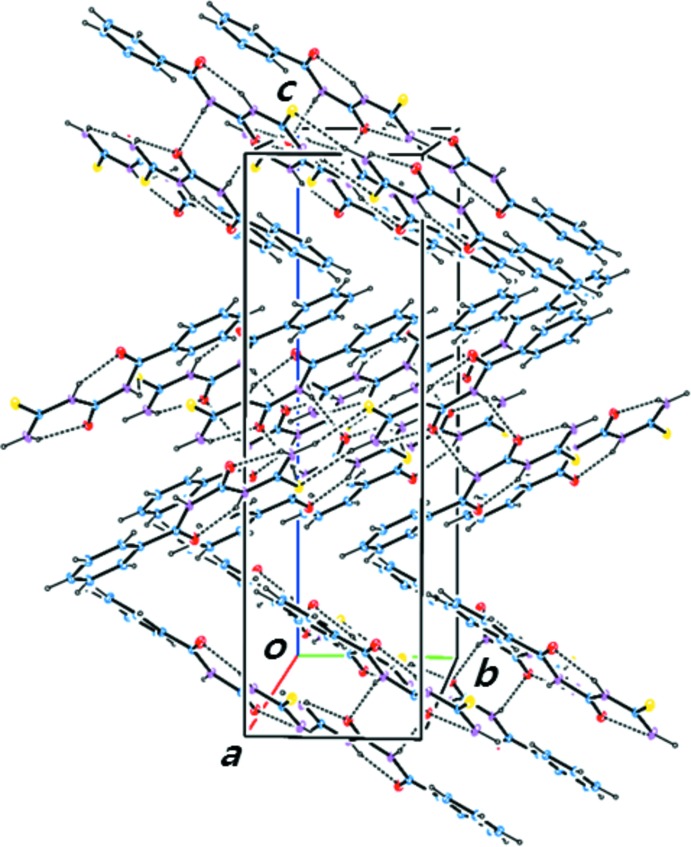
Part of the packing diagram of (I), showing molecules linked by intermolecular N—H···O and N—H···S hydrogen bonds (dashed lines).

## supplementary crystallographic information

### 1. Comment

Derivatives of 5-amino-2*H*-1,2,4-thiadizolin-3-one have arrested the attention on the antibacterial activity, potential carcinogenicity, and kinase inhibitor activity (Piskala *et al.*, 2004; Castro *et al.*, 2008). As a part of our continuous interest in the synthesis of novel potential anti-metabolites of nucleic acid components which would possess cytostatic activity, we have synthesized derivatives of 5-amino-3*H*-1,3,4-thiadiazol-2-one (Cho *et al.*, 1996). The title compound, 1-benzoyl-4-thiobiuret is an isomer of 1-benzoyl-2-thiobiuret (Kang *et al.*, 2012). This compound is an intermediate for the formation of the thiobiuret which is a good starting material to make 5-amino-2*H*-1,2,4-thiadizolin-3-one *via* oxidative ring closure reaction.

In (I), Fig. 1, the dihedral angle between the benzoyl unit (C1 —C7/O8 atoms, r.m.s. deviation = 0.068 Å) and thiobiuret group (N9 — N15 atoms) is 9.67 (13) °. The carbonyl-O8 and O11 atoms are positioned *anti* to each other, and S14 atom is also positioned *anti* to carbonyl-O11 atom. The molecular structure is stabilized by two intramolecular N—H···O hydrogen bonds (Fig. 1 and Table 1). In the crystal packing, the intermolecular N—H···O and N—H···S interactions link the molecules into zigzag chains extending along the *c* axis (Fig. 2).

### 2. Experimental

Benzoyl chloride (48 ml, 58.1 g, 0.41mole) was added to warm solution of potassium thiocyanate (48.0 g, 0.49mole) in acetone (400 ml). The solution became milky white and yellow when the addition had been completed. The mixture was stirred for 3.5 h at 50 °C and left to cool to room temperature. The filtrate was heated to 55 °C for 5 h with urea (24.0 g, 0.40 mole). And the resulting solution was cooled to room temperature and then placed in an ice bath for several hours. The cold mixture was filtered to give 1-benzoyl-4-thiobiuret as a bright yellow solid. The title compound (I) was obtained after recrystallization from its acetonitrile-methanol (10: 1) solution.

### 3. Refinement

H atoms of the NH and NH_2_ groups were located in a difference Fourier map and refined freely [refined distances = 0.81 (3) – 0.98 (4) Å]. Other H atoms were positioned geometrically and refined using a riding model, with C—H = 0.93 Å, and with *U*_iso_(H) = 1.2*U*_eq_(C).

### Crystal data

**Table:**

C_9_H_9_N_3_O_2_S	*F*(000) = 928
*M**_r_* = 223.25	*D*_x_ = 1.435 Mg m^−^^3^
Monoclinic, *C*2/*c*	Mo *K*α radiation, λ = 0.71073 Å
Hall symbol: -C 2yc	Cell parameters from 883 reflections
*a* = 14.4259 (14) Å	θ = 2.8–20.7°
*b* = 6.6145 (6) Å	µ = 0.30 mm^−^^1^
*c* = 21.722 (2) Å	*T* = 296 K
β = 94.166 (3)°	Block, yellow
*V* = 2067.2 (3) Å^3^	0.18 × 0.12 × 0.04 mm
*Z* = 8	

### Data collection

**Table:**

Bruker SMART CCD area-detector diffractometer	1293 reflections with *I* > 2σ(*I*)
Graphite monochromator	*R*_int_ = 0.059
φ and ω scans	θ_max_ = 26.0°, θ_min_ = 1.9°
Absorption correction: multi-scan (*SADABS*; Bruker, 2002)	*h* = −14→17
*T*_min_ = 0.96, *T*_max_ = 0.99	*k* = −8→3
7756 measured reflections	*l* = −26→14
1973 independent reflections	

### Refinement

**Table:**

Refinement on *F*^2^	0 restraints
Least-squares matrix: full	Hydrogen site location: mixed
*R*[*F*^2^ > 2σ(*F*^2^)] = 0.047	H atoms treated by a mixture of independent and constrained refinement
*wR*(*F*^2^) = 0.115	*w* = 1/[σ^2^(*F*_o_^2^) + (0.0483*P*)^2^ + 0.5497*P*] where *P* = (*F*_o_^2^ + 2*F*_c_^2^)/3
*S* = 1.03	(Δ/σ)_max_ = 0.001
1973 reflections	Δρ_max_ = 0.21 e Å^−^^3^
152 parameters	Δρ_min_ = −0.23 e Å^−^^3^

### Special details

**Table:**

Geometry. All e.s.d.'s (except the e.s.d. in the dihedral angle between two l.s. planes) are estimated using the full covariance matrix. The cell e.s.d.'s are taken into account individually in the estimation of e.s.d.'s in distances, angles and torsion angles; correlations between e.s.d.'s in cell parameters are only used when they are defined by crystal symmetry. An approximate (isotropic) treatment of cell e.s.d.'s is used for estimating e.s.d.'s involving l.s. planes.

### Fractional atomic coordinates and isotropic or equivalent isotropic displacement parameters (Å^2^)

**Table:**

	*x*	*y*	*z*	*U*_iso_*/*U*_eq_	
C1	0.33533 (17)	1.0358 (4)	0.65485 (12)	0.0376 (6)	
C2	0.3931 (2)	1.1557 (4)	0.69320 (13)	0.0488 (7)	
H2	0.4542	1.1162	0.7031	0.059*	
C3	0.3604 (2)	1.3340 (4)	0.71691 (15)	0.0589 (9)	
H3	0.3994	1.4133	0.7429	0.071*	
C4	0.2704 (2)	1.3943 (4)	0.70214 (14)	0.0573 (9)	
H4	0.2492	1.5159	0.7174	0.069*	
C5	0.2120 (2)	1.2761 (4)	0.66512 (15)	0.0563 (8)	
H5	0.1508	1.3161	0.6559	0.068*	
C6	0.24417 (19)	1.0959 (4)	0.64118 (13)	0.0486 (7)	
H6	0.2043	1.0158	0.616	0.058*	
C7	0.37532 (18)	0.8447 (4)	0.63138 (13)	0.0396 (7)	
O8	0.44757 (13)	0.7726 (3)	0.65381 (9)	0.0561 (6)	
N9	0.32479 (17)	0.7544 (3)	0.58270 (11)	0.0439 (6)	
H9	0.2806 (18)	0.812 (4)	0.5652 (12)	0.039 (8)*	
C10	0.34054 (18)	0.5751 (4)	0.55260 (13)	0.0384 (6)	
O11	0.28343 (12)	0.5116 (3)	0.51289 (9)	0.0472 (5)	
N12	0.42172 (15)	0.4788 (3)	0.57068 (11)	0.0393 (6)	
H12	0.4561 (17)	0.535 (4)	0.6002 (12)	0.037 (8)*	
C13	0.45494 (17)	0.3010 (4)	0.54659 (13)	0.0382 (7)	
S14	0.55856 (5)	0.21601 (11)	0.57483 (4)	0.0510 (3)	
N15	0.40313 (19)	0.2107 (4)	0.50312 (13)	0.0521 (7)	
H15A	0.425 (2)	0.083 (5)	0.4869 (15)	0.086 (11)*	
H15B	0.351 (2)	0.248 (5)	0.4927 (15)	0.068 (11)*	

### Atomic displacement parameters (Å^2^)

**Table:**

	*U*^11^	*U*^22^	*U*^33^	*U*^12^	*U*^13^	*U*^23^
C1	0.0416 (16)	0.0347 (14)	0.0363 (16)	−0.0008 (11)	0.0021 (12)	−0.0012 (12)
C2	0.0482 (18)	0.0489 (17)	0.0488 (19)	−0.0032 (13)	0.0008 (14)	−0.0116 (15)
C3	0.071 (2)	0.0495 (18)	0.056 (2)	−0.0128 (16)	0.0079 (17)	−0.0189 (16)
C4	0.076 (2)	0.0399 (17)	0.057 (2)	0.0053 (15)	0.0172 (17)	−0.0096 (15)
C5	0.058 (2)	0.0545 (19)	0.057 (2)	0.0191 (15)	0.0040 (15)	−0.0054 (16)
C6	0.0497 (18)	0.0467 (17)	0.0487 (19)	0.0032 (13)	−0.0009 (14)	−0.0070 (14)
C7	0.0392 (17)	0.0348 (14)	0.0440 (18)	0.0001 (11)	−0.0025 (13)	−0.0001 (13)
O8	0.0495 (13)	0.0491 (12)	0.0667 (15)	0.0115 (10)	−0.0165 (11)	−0.0113 (10)
N9	0.0457 (15)	0.0362 (13)	0.0476 (16)	0.0126 (11)	−0.0111 (12)	−0.0071 (11)
C10	0.0351 (15)	0.0319 (14)	0.0476 (18)	0.0022 (11)	−0.0005 (13)	−0.0022 (13)
O11	0.0416 (11)	0.0413 (11)	0.0568 (13)	0.0059 (9)	−0.0097 (10)	−0.0098 (10)
N12	0.0349 (13)	0.0330 (12)	0.0488 (16)	0.0047 (10)	−0.0054 (11)	−0.0070 (11)
C13	0.0353 (15)	0.0312 (13)	0.0486 (18)	0.0004 (11)	0.0059 (13)	−0.0012 (13)
S14	0.0368 (4)	0.0439 (4)	0.0713 (6)	0.0082 (3)	−0.0014 (4)	−0.0077 (4)
N15	0.0417 (17)	0.0421 (15)	0.071 (2)	0.0092 (12)	−0.0036 (14)	−0.0195 (13)

### Geometric parameters (Å, º)

**Table:**

C1—C2	1.384 (4)	C7—O8	1.216 (3)
C1—C6	1.385 (3)	C7—N9	1.376 (3)
C1—C7	1.494 (3)	N9—C10	1.381 (3)
C2—C3	1.383 (4)	N9—H9	0.81 (3)
C2—H2	0.93	C10—O11	1.223 (3)
C3—C4	1.373 (4)	C10—N12	1.366 (3)
C3—H3	0.93	N12—C13	1.386 (3)
C4—C5	1.367 (4)	N12—H12	0.86 (3)
C4—H4	0.93	C13—N15	1.306 (4)
C5—C6	1.393 (4)	C13—S14	1.671 (3)
C5—H5	0.93	N15—H15A	0.98 (4)
C6—H6	0.93	N15—H15B	0.81 (3)
			
C2—C1—C6	119.0 (2)	O8—C7—N9	121.9 (2)
C2—C1—C7	117.4 (2)	O8—C7—C1	122.4 (2)
C6—C1—C7	123.6 (2)	N9—C7—C1	115.7 (2)
C3—C2—C1	120.4 (3)	C7—N9—C10	129.8 (2)
C3—C2—H2	119.8	C7—N9—H9	120.5 (19)
C1—C2—H2	119.8	C10—N9—H9	109.4 (19)
C4—C3—C2	120.2 (3)	O11—C10—N12	124.2 (2)
C4—C3—H3	119.9	O11—C10—N9	120.2 (2)
C2—C3—H3	119.9	N12—C10—N9	115.6 (2)
C5—C4—C3	120.2 (3)	C10—N12—C13	126.9 (2)
C5—C4—H4	119.9	C10—N12—H12	116.6 (17)
C3—C4—H4	119.9	C13—N12—H12	116.5 (17)
C4—C5—C6	120.1 (3)	N15—C13—N12	117.7 (2)
C4—C5—H5	120	N15—C13—S14	124.1 (2)
C6—C5—H5	120	N12—C13—S14	118.1 (2)
C1—C6—C5	120.1 (3)	C13—N15—H15A	118.2 (18)
C1—C6—H6	119.9	C13—N15—H15B	122 (2)
C5—C6—H6	119.9	H15A—N15—H15B	119 (3)

### Hydrogen-bond geometry (Å, º)

**Table:**

*D*—H···*A*	*D*—H	H···*A*	*D*···*A*	*D*—H···*A*
N9—H9···O11^i^	0.81 (3)	2.20 (3)	2.945 (3)	151 (2)
N12—H12···O8	0.86 (3)	1.97 (3)	2.661 (3)	136 (2)
N15—H15*A*···S14^ii^	0.98 (4)	2.41 (4)	3.358 (3)	163 (3)
N15—H15*B*···O11	0.81 (3)	2.06 (3)	2.653 (3)	130 (3)

Symmetry codes: (i) −*x*+1/2, −*y*+3/2, −*z*+1; (ii) −*x*+1, −*y*, −*z*+1.
